# Implications of empirical administration of caspofungin in COVID-19 complicated fungal infections

**DOI:** 10.3389/fcimb.2023.1269543

**Published:** 2023-11-21

**Authors:** Kazuhiro Itoh, Hiroshi Tsutani, Yasuhiko Mitsuke, Hiromichi Iwasaki

**Affiliations:** ^1^ Department of Internal Medicine, NHO Awara National Hospital, Awara, Fukui, Japan; ^2^ Division of Infection Control and Prevention, University of Fukui Hospital, Fukui, Japan

**Keywords:** COVID-19, SARS-CoV-2, co-infection, fungal infections, caspofungin, spleen tyrosine kinase

## SARS-CoV-2 infection and fungal infections

*Candida* and *Aspergillus* spp. were identified as the most common causes of invasive fungal infections in COVID-19 patients (Ezeokoli and Pohl, 2020). The mortality rate of invasive Candida infections was twice as high in COVID-19 patients as in non-COVID-19 patients (Seagle et al., 2022). Therefore, prevention of the complications of fungal infections in COVID-19 is a crucial issue with implications for prognosis.

## Risk factors for COVID-19-associated candidiasis

Yazdanpanah S et al. analyzed risk factors for COVID-19-associated candidiasis (Yazdanpanah et al., 2023). They used multiple logistic regression to show that heart failure, bacterial co-infection, and empiric antifungal use were significant risk factors for *Candida* co-infection in hospitalized COVID-19 patients (Yazdanpanah et al., 2023). In their study, azoles were used for empirical antifungal treatment, and in some cases, azoles were used in combination with the echinocandin antifungal agent caspofungin. The antifungal effects seem to differ among these drugs. While some *Candida* strains were resistant to azoles, caspofungin was effective against all isolated strains in that study (Yazdanpanah et al., 2023). In addition, the study found higher odds of in-hospital mortality in *Candida* culture-positive patients (RR: 2.18, 95% CI: 0.9, 5.2) (Yazdanpanah et al., 2023), but no subgroup analysis classified by empiric therapy was performed. We believe that this study should elucidate whether empirical caspofungin treatment can improve prognosis in a cohort of COVID-19 patients with risk factors for fungal infection complications.

## Chemical properties of caspofungin

**Figure 1:**
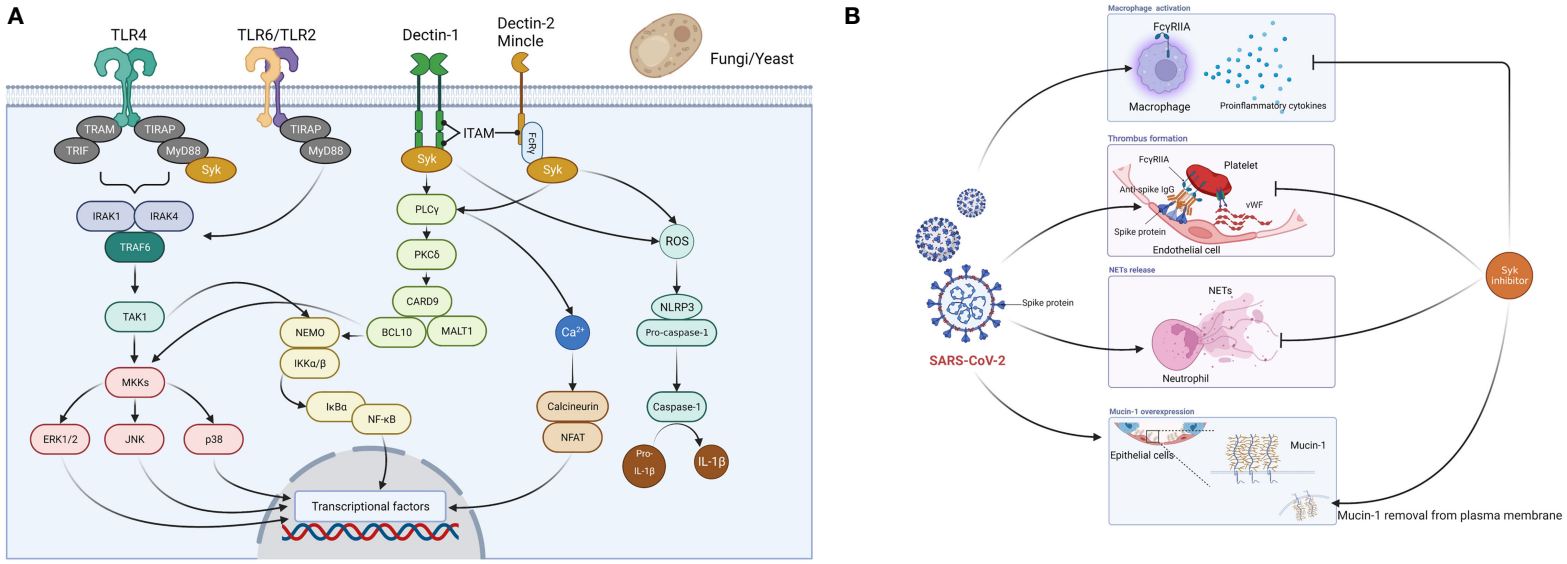
Spleen tyrosine kinase (Syk)-mediated mechanism of action of caspofungin. **(A)** Fungi (yeast-like fungi) are recognized by PRRs (TLR2, TLR4, TLR6, Dectin-1, Dectin-2, Mincle) expressed on host immune cells such as monocytes and macrophages. When pathogens are recognized by Dectin-1, phosphorylation of immunoreceptor tyrosine-based activation motif (ITAM)-like motifs occur and Syk is recruited. There are three major pathways downstream of the Syk signaling pathway. First, several proteins, including phospholipase C-γ (PLCγ), protein kinase C-δ (PKCδ), caspase recruitment domain-containing protein 9 (CARD9), B-cell lymphoma 10 (BCL10), and mucosa-associated lymphoid tissue lymphoma translocation protein 1 (MALT1) activate NF-kB essential modulator (NEMO) and IkB kinase (IKK) complexes, which then induce NF-kB signaling. This subsequently triggers the 3MAPK (p38, JNK, ERK1/2) pathway and induces activator protein 1 expression. Second, PLCγ induces an increase in Ca2+ and triggers the calcineurin and NFAT pathways. Third, Syk-dependent reactive oxygen species (ROS) production activates the nucleotide-binding domain and leucine-rich repeat-containing protein 3 (NLRP3) inflammasome, which triggers caspase-1 activation and cleaves pro-IL-1b to generate IL-1b. When Dectin-2 and Mincle cooperate with the Fc-γ receptor, Syk is recruited to ITAM and triggers PKCδ-CARD9-dependent NF-kB activation. TLR2/TLR6 heterodimers recruit MyD88 protein and activate a series of kinases [IL-1 receptor-related kinase (IRAK)1, IRAK4, TNF receptor-associated factor 6 (TRAF6), and transforming growth factor-β-activated kinase 1 (TAK1)] to phosphorylate the NEMO-IKK complex, which subsequently triggers NF-kB. Fungal (mannan) recognition by TLR4 is a relatively minor signaling pathway, and the major ligand for TLR4 is LPS. TLR4 associates with MyD88 via its intracellular domain and activates IRAK, which activates TRAF6 and the MAPK and IkBα pathways. Syk cooperates with TLR4 and the adaptor molecule MyD88 and plays an important role in LPS-triggered signaling. These cascades ultimately activate the production of inflammatory cytokines and chemokines. **(B)** Syk inhibitors suppress macrophage activation and overproduction of proinflammatory cytokines, thrombus formation, and the release of neutrophil extracellular traps (NETs) caused by SARS-CoV-2, and remove overexpressed mucin-1 from the cell membrane in lung epithelial cells. PRRs, pattern recognition receptors; TIR, Toll/interleukin-1 receptor; TIRAP, TIR domain-containing adaptor protein; TLR, toll-like receptor; TRAM, TRIF-related adaptor molecule; TRIF, TIR domain-containing adaptor protein inducing interferon-β. Figures created with BioRender (https://biorender.com/).

Caspofungin is an echinocandin antifungal drug that exerts its antifungal activity by targeting β-D-glucan synthase in the membrane of fungal cells, an enzyme that is absent in the human body (Patil and Majumdar, 2017). The mechanism by which caspofungin inhibits the main protease of SARS-CoV-2 suggests that echinocandin-based antifungals including caspofungin inhibit the intracellular viral replication process of SARS-CoV-2 (Liu and Wang, 2020; Nakajima et al., 2023). In addition to these effects, our research has shown host immunomodulatory effects (Itoh et al., 2021). Caspofungin inhibited inflammatory cytokine and chemokine production in THP-1 cells stimulated with LPS or zymosan. In this model, investigation of signaling pathways revealed that caspofungin inhibited the activation of spleen tyrosine kinase (Syk) and its downstream signaling molecules (**Figure 1A**) (Itoh et al., 2021). Binding to the ATP-binding site of Syk and inhibition of its kinase activity as an ATP-competitive inhibitor, or inhibition of the association of Syk with a substrate protein, is generally accepted as the mechanism of Syk inhibition. However, the mechanism by which caspofungin inhibits Syk has not yet been elucidated. Additionally, it has not been determined whether or not caspofungin acts on signaling pathways other than those involving Syk. Immune cell receptors involved in Syk activation include C-type lectin receptors (Dectin-1, Dectin-2, Mincle) and toll-like receptor (TLR) 4 (Chaudhary et al., 2007; Mócsai et al., 2010). The TLR 4-mediated signaling pathway has been found to be activated in COVID-19 patients (Sohn et al., 2020), and thus inhibition of the Syk pathway may suppress the overproduction of proinflammatory cytokines.

## Syk inhibitors’ utility against COVID-19

Here we summarize the relationship between COVID-19 and Syk inhibitors. R406, a Syk inhibitor, inhibits both the mainly FcγRIIA-dependent release of proinflammatory cytokines by macrophages and thrombus formation induced by the anti-spike immune complex (Bye et al., 2021; Hoepel et al., 2021). R406 inhibits the release of neutrophil extracellular traps (NETs) when healthy neutrophils are stimulated with plasma from COVID-19 patients (Strich et al., 2021). NETs have been found in the lungs of deceased COVID-19 patients and are promoters of immune thrombosis (Papayannopoulos, 2018; Middleton et al., 2020; Veras et al., 2020). Furthermore, NETs are associated with COVID-19 severity (Zuo et al., 2020). R406 also inhibits mucin-1, a transmembrane protein of the lung epithelium associated with acute respiratory distress syndrome (ARDS) (Kost-Alimova et al., 2020) (**Figure 1B**). Caspofungin, which inhibits the Syk signaling pathway, may therefore reduce the severity of COVID-19 via the same mechanism described prior.

## Caspofungin’s efficacy in COVID-19 cytokine storm models (*in vitro* and *in vivo*)

In a SARS-CoV-2-specific chimeric antigen receptor (CAR)-T-cell model established to mimic the cytokine storm in COVID-19 patients, caspofungin suppressed inflammatory cytokine production (Xia et al., 2023). It also suppressed lethal inflammation, ameliorated severe pneumonia, and reduced mortality in a SARS-CoV-2-infected Syrian hamster model (Xia et al., 2023). T lymphocytes generally do not express Syk in their T cell receptors (TCRs) but do express ZAP-70, which contributes to T cell activation (Mócsai et al., 2010). However, Syk inhibitors (R406 and GS-9973) suppress TCR-stimulated phosphorylation of ZAP-70 (Colado et al., 2017). Thus, we believe that the inhibitory effect of caspofungin on Syk suppressed the COVID-19 cytokine storm in *in vitro* and *in vivo* models.

## Conclusion

Caspofungin is an antifungal agent with activity against *Candida* and *Aspergillus*, which are common pathogens associated with COVID-19 (Ezeokoli and Pohl, 2020). Caspofungin inhibits the proliferation of SARS-CoV-2 directly (Liu and Wang, 2020; Nakajima et al., 2023). Additionally, caspofungin has an inhibitory effect on host immune cells against Syk (Itoh et al., 2021), which promotes the suppression of COVID-19 severity by inhibiting inflammatory cytokines, immunothrombosis, and ARDS. Moreover, caspofungin has fewer side effects than azoles and amphotericin B because it targets the β-D-glucan synthase, which is not generally found in the human body (Patil and Majumdar, 2017). Because fungal infections can be difficult to diagnose, even with limited tests (antigen and culture tests), and because treatment of patients with fungal infection complications can be delayed, leading to severe disease, patients at risk must receive empirical administration (early administration). On the other hand, there is a risk of overtreating COVID-19 patients who are not at high risk for fungal infections, which could expose patients to unnecessary drug-related adverse effects (mostly liver dysfunction) and increased drug cost burden. In conclusion, we believe that empirical administration of caspofungin to COVID-19 patients may prevent worsening of prognosis by preventing fungal infection complications in addition to reducing the severity of COVID-19, but is especially recommended in hospitalized COVID-19 patients with risk factors for fungal infection complications, such as heart failure and bacterial co-infection. Further studies are warranted to clarify the prognostic benefit of empiric administration of caspofungin to COVID-19 patients.

## Author contributions

KI: Conceptualization, Data curation, Formal Analysis, Investigation, Methodology, Project administration, Resources, Validation, Visualization, Writing – original draft, Writing – review & editing. HT: Conceptualization, Methodology, Supervision, Validation, Writing – original draft, Writing – review & editing. YM: Conceptualization, Methodology, Supervision, Validation, Writing – original draft, Writing – review & editing. HI: Conceptualization, Methodology, Project administration, Supervision, Validation, Writing – original draft, Writing – review & editing.
